# Tolfenamic Acid Derivatives: A New Class of Transcriptional Modulators with Potential Therapeutic Applications for Alzheimer’s Disease and Related Disorders

**DOI:** 10.3390/ijms242015216

**Published:** 2023-10-16

**Authors:** Jaunetta Hill, Karim E. Shalaby, Syed W. Bihaqi, Bothaina H. Alansi, Benjamin Barlock, Keykavous Parang, Richard Thompson, Khalid Ouararhni, Nasser H. Zawia

**Affiliations:** 1 Department of Biomedical and Pharmaceutical Sciences, College of Pharmacy, University of Rhode Island, Kingston, RI 02881, USA; jaunettahill@uri.edu (J.H.); syedwasim7@gmail.com (S.W.B.); bothainaalansi@gmail.com (B.H.A.); bbarlock@uri.edu (B.B.); 2 Neurological Disorder Research Center, Qatar Biomedical Research Institute (QBRI), Hamad Bin Khalifa University (HBKU), Qatar Foundation, Doha 34110, Qatar; karimshalaby@hbku.edu.qa (K.E.S.); kouararhni@hbku.edu.qa (K.O.); 3 Center for Targeted Drug Delivery, Department of Biomedical and Pharmaceutical Sciences, Chapman University School of Pharmacy, Harry and Diane Rinker Health Science Campus, Irvine, CA 92618, USA; parang@chapman.edu; 4 George and Anne Ryan Institute for Neuroscience, University of Rhode Island, Kingston, RI 02881, USA; 5 Interdisciplinary Neuroscience Program, University of Rhode Island, Kingston, RI 02881, USA; 6 Biological and Biomedical Sciences Division, College of Health & Life Sciences (CHLS), Hamad Bin Khalifa University (HBKU), Qatar Foundation, Doha 34110, Qatar

**Keywords:** Alzheimer’s disease, drug design, drug development, small molecules, tolfenamic acid, SP1

## Abstract

The field of Alzheimer’s disease (AD) has witnessed recent breakthroughs in the development of disease-modifying biologics and diagnostic markers. While immunotherapeutic interventions have provided much-awaited solutions, nucleic acid-based tools represent other avenues of intervention; however, these approaches are costly and invasive, and they have serious side effects. Previously, we have shown in AD animal models that tolfenamic acid (TA) can lower the expression of AD-related genes and their products and subsequently reduce pathological burden and improve cognition. Using TA as a scaffold and the zinc finger domain of SP1 as a pharmacophore, we developed safer and more potent brain-penetrating analogs that interfere with sequence-specific DNA binding at transcription start sites and predominantly modulate the expression of SP1 target genes. More importantly, the proteome of treated cells displayed ~75% of the downregulated products as SP1 targets. Specific levels of SP1-driven genes and AD biomarkers such as amyloid precursor protein (APP) and Tau proteins were also decreased as part of this targeted systemic response. These small molecules, therefore, offer a viable alternative to achieving desired therapeutic outcomes by interfering with both amyloid and Tau pathways with limited off-target systemic changes.

## 1. Introduction

There are currently over 50 million people worldwide living with dementia, mostly due to AD, with an expected doubling every 20 years. The recent US Food and Drug Administration’s accelerated approvals of aducanumab and lecanemab provide the first disease-modifying treatments that target the accumulation of amyloid plaques; however, they do not target Tau pathology. Therefore, more disease-modifying treatments are needed to prevent or slow the progression of neurodegenerative diseases by addressing both amyloid and Tau pathologies.

**Figure 1:**
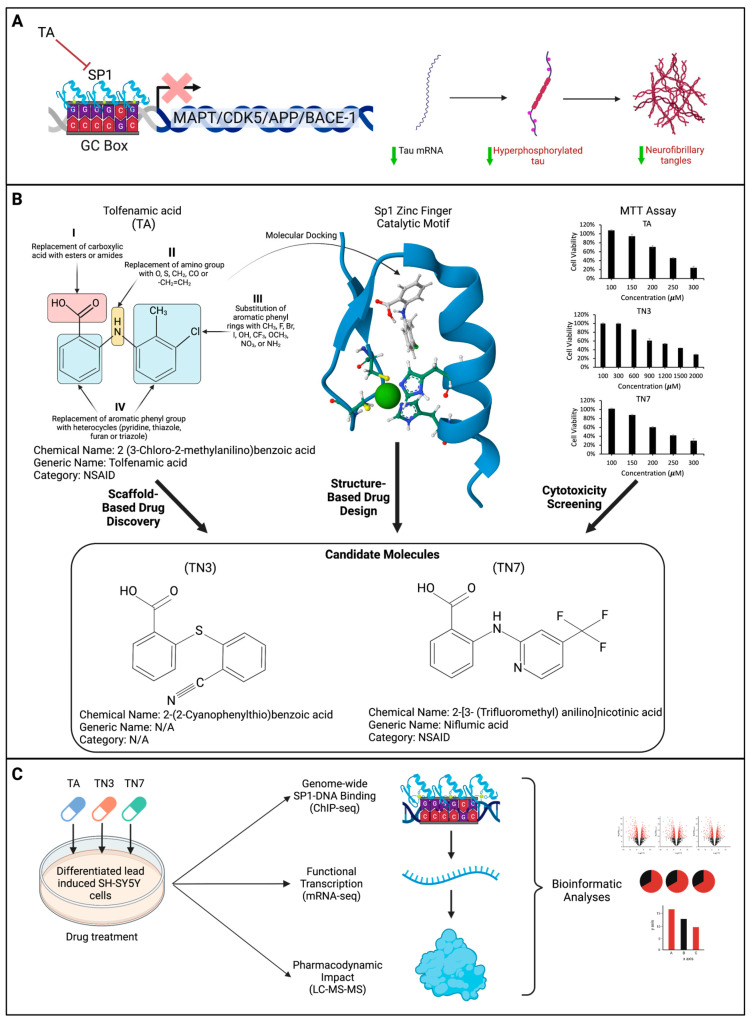
Schematic of the proposed mechanism of action of tolfenamic acid: (**A**) Tolfenamic acid (TA) inhibits specificity protein 1 (SP1)-DNA binding, which may lead to decreased transcription of genes associated with Alzheimer’s disease such as microtubule-associated protein Tau (MAPT), cyclin-dependent kinase-5 (CDK5), amyloid precursor protein (APP), and β-secretase-1 (BACE-1). The illustration shows the impact of TA on Tau tangles as a consequence of inhibiting SP1-DNA binding. (**B**) This schematic illustrates the procedure of designing and screening candidate drugs via scaffold-based drug design, structure-based drug discovery, and cytotoxicity screening. The core structure of TA and the various changes (I-IV) that were employed to synthesize new compounds through scaffold-based drug discovery are shown. SP1’s zinc finger 1 catalytic motif (PDB: 1VA1) was used as a model for molecular docking experiments. A viability MTT assay was employed to evaluate the cytotoxicity of the synthesized compounds, and two candidate drugs (TN3 and TN7) were selected based on their safety profiles. The cytotoxicity screening results of TA and analogs in SH-SY5Y cells are presented. Differentiated SH-SY5Y cells were exposed to 25 µM of lead acetate for 48 h to induce SP1 expression. Subsequently, the cells were treated with TA, TN3, and TN7 at various concentrations (as specified) for 72 h, and an MTT assay was performed to assess the cytotoxicity of each small molecule. Data are expressed as ± S.E.M; n was 3 for each treatment group. (**C**) A schematic representation of the multiomic study design (ChIP-sequencing, mRNA-sequencing, and Liquid Chromatography Tandem Mass Spectrometry) for the assessment of the mode of action of TA, TN3, and TN7 in differentiated and lead-induced SH-SY5Y cells.

Fenamate nonsteroidal anti-inflammatory drugs (NSAIDs) have been implicated in several noncanonical pathways related to neuropathology [1]. Of the fenamate NSAIDs, tolfenamic acid (TA) is a well-tolerated small molecule commonly prescribed for migraine headaches in Europe under the common name Clotam Rapid. TA has been proven to lower the total Tau, phosphorylated Tau, and amyloid precursor protein (APP) in human transgenic mice via a mechanism involving specificity protein 1 (SP1) [2,3,4]. SP1 is a transcription factor involved in the regulation of multiple AD-related genes (Figure 1A).

SP1 has a unique property that can modulate the two hallmark features of AD, namely, amyloid plaques and Tau tangles. The 5′-flanking regulatory region of the APP gene is rich in GC box elements and contains consensus sites that are recognized by several transcription factors, including SP1 [5,6]. SP1 binds to both the human and rat APP promoters and accelerates the production of APP mRNA, which can be further spliced to generate several cell-specific species [7,8,9]. SP1 belongs to a family of zinc finger protein (ZFP) transcription factors and is elevated in AD patients [10,11]. Furthermore, SP1 regulates the expression of BACE1; the APP processing enzyme; and Tau, the main component of tangles [12]. The buildup of hyperphosphorylated Tau results in the formation of the pathogenic tangles found in AD and some other neurodegenerative disorders, such as progressive supranuclear palsy [13,14].

The pharmacokinetics of TA are favorable for neurodegenerative disease, as research has shown that it crosses the blood–brain barrier (BBB) [15]. Given the efficacy of TA in reducing APP and Tau levels in APP and Tau transgenic mouse models [2,3,4], along with its established clinical use and safety profile, it serves as a promising lead compound for the design and synthesis of improved drug candidates (Figure 1B). Moreover, the demonstration of TA’s interaction with the zinc finger domain of SP1 provides further motivation for exploring the structure–activity profile of TA analogs for the downregulation of AD-related pathways.

In this study, we aimed to analyze the genome-wide differential binding of SP1 and determine the ability of these compounds to modulate it using ChIP-Seq (chromatin immunoprecipitation sequencing) technology (Figure 1C). We then monitored the consequential impact on global mRNA expression and the on-target and off-target proteomic profiles following treatment with TA and its derivatives TN3 and TN7 in an in vitro model, as well as alterations in key biomarkers associated with AD (Figure 1C).

## 2. Results

### 2.1. Derivatives of Tolfenamic Acid (TA)

TA has been identified as a promising disease-modifying drug candidate based on its established activity on APP and Tau downregulation and the effects it has demonstrated in disease models, in addition to its clinical use and safety profile [2,3,4]. Hence, TA serves as an ideal lead compound for designing TA derivatives and analogs to explore the structure–activity relationships (SARs) of APP and Tau downregulation.

**Figure 2:**
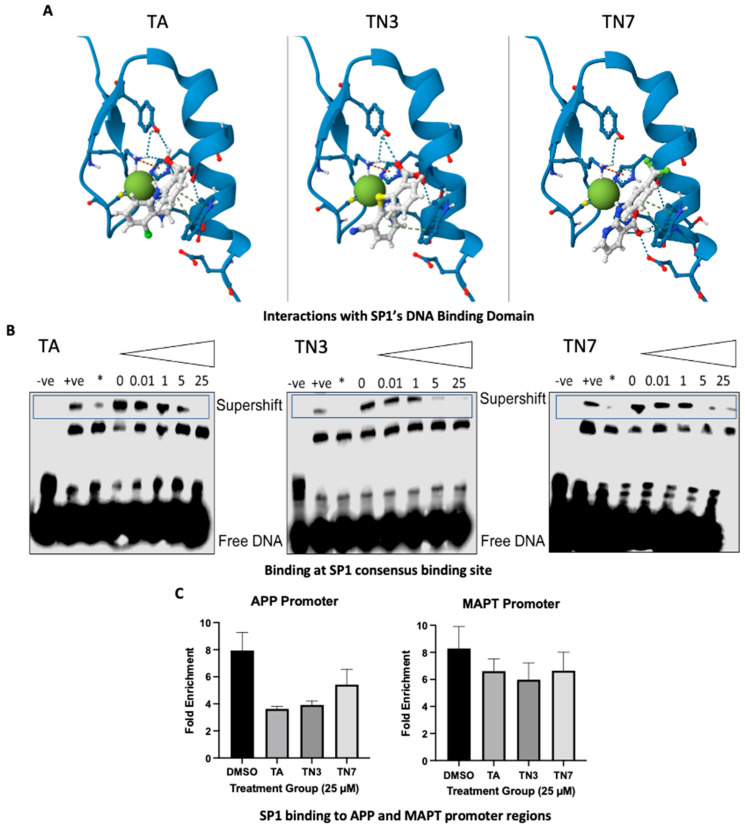
TA, TN3, and TN7 interfere with SP1-DNA binding to its genomic binding site: (**A**) Molecular docking of TA and its derivatives. Ligand interaction diagrams for the molecular docking complexes between transcription factor SP1-DNA binding domain (zinc finger 1) (PDB ID: 1VA1 [19]) and TA and derivatives with favorable interaction energies and conformational reorganization in the catalytic motif. Differentiated SH-SY5Y cells were exposed to 25 µM of lead acetate for 48 h and were subsequently treated with 25 µM of TA, TN3, or TN7 for 72 h. (**B**) Electrophoretic mobility shift assay (EMSA) using protein lysates of TA/TN3/TN7-treated cells and biotinylated SP1 oligos containing SP1 consensus genomic binding sequence; ‘−ve’ indicates probe-only negative control ‘+ve’ indicates labeled probe and total protein lysate positive control; * indicates labeled probe, total protein lysates, and 200-fold molar excess of unlabeled probe. Triangle indicates increased concentrations (0, 0.01, 1, 5, and 25 µM). (**C**) ChIP-qPCR analysis of APP and MAPT, respectively. Data are expressed as ± S.E.M. and statistical significance was determined using a one-way ANOVA followed by Dunnett’s test for multiple comparisons; n was between 4 and 6 for each treatment group. (* *p* < 0.05).

SARs were established and optimized for TA analogs through a systematic design and synthesis approach. Molecular modeling studies were conducted to identify four regions in TA that could be modified (Figure 1B). Initial molecular orbital calculations and geometry optimizations were performed to study the effect of functional group modifications on the conformation of TA. Subsequently, the binding affinity of the compounds to zinc ions was evaluated, considering interaction energy and conformational reorganization. These measures guided the selection of specific compounds for further exploration. Furthermore, the inhibition of DNA binding of SP1 by TA and its derivatives was examined. A model of SP1 was prepared by adding hydrogen atoms and identifying the catalytic motif C2H2. The compounds were docked near the zinc ion and the catalytic motif, and the interaction energy was calculated (Figure 2A). The results revealed that 10 selected compounds displayed favorable interaction and binding affinity for the zinc finger domain of SP1, indicating their potential as effective inhibitors of DNA binding.

### 2.2. Cytotoxicity Screening of TA and Analogs in SH-SY5Y Cells

Differentiated SH-SY5Y cells were treated with TA and its derivatives for 72 h at various concentrations (Figure 1B). Eight compounds that demonstrated higher cytotoxicity than TA were eliminated from this study. Based on their superior safety profiles, we selected two compounds for further testing: the non-fenamate 2-(2-Cyanophenylthio)benzoic acid (TN3) and the fenamate niflumic acid (TN7). Our analysis showed that TN7 was as safe as TA, and TN3 was the least cytotoxic (Figure 1B).

### 2.3. Loss of SP1-DNA Binding and Genomic Interaction

Previous research both in the brain and other tissues has shown that TA can impact SP1 protein levels but not its gene expression [16,17]. Furthermore, earlier reports for TA in non-neural tissue confirmed that TA treatment prevented SP1 binding to its consensus genomic binding element [17]. To explore whether the analogs followed the same mechanism, we first investigated the target engagement of each drug using EMSA analysis (Figure 2B). Lysates of SH-SY5Y cells that were treated with TA, TN3, and TN7 for 72 h at 0–25 µM were incubated with SP1’s consensus genomic binding element (5′- GCTCGCCCCGCCCCGATCGAAT-3′). The analysis revealed that TA, TN3, and TN7 decreased protein–DNA binding (Figure 2B). TA decreased binding at 25 µM, while TN3 and TN7 appeared to decrease binding at 5 and 25 µM. 

To verify this interaction, we used ChIP-qPCR using an SP1-specific antibody to pull down promoter regions of putative AD-related target genes. Our results demonstrated that treatment TA or its analogs showed a trend toward decreased SP1-DNA binding to the promoter regions of APP and Tau genes (Figure 2C). These data are consistent with the report that siRNA silencing and the depletion of SP1 significantly reduced APP promoter responsiveness [18]. 

**Figure 3:**
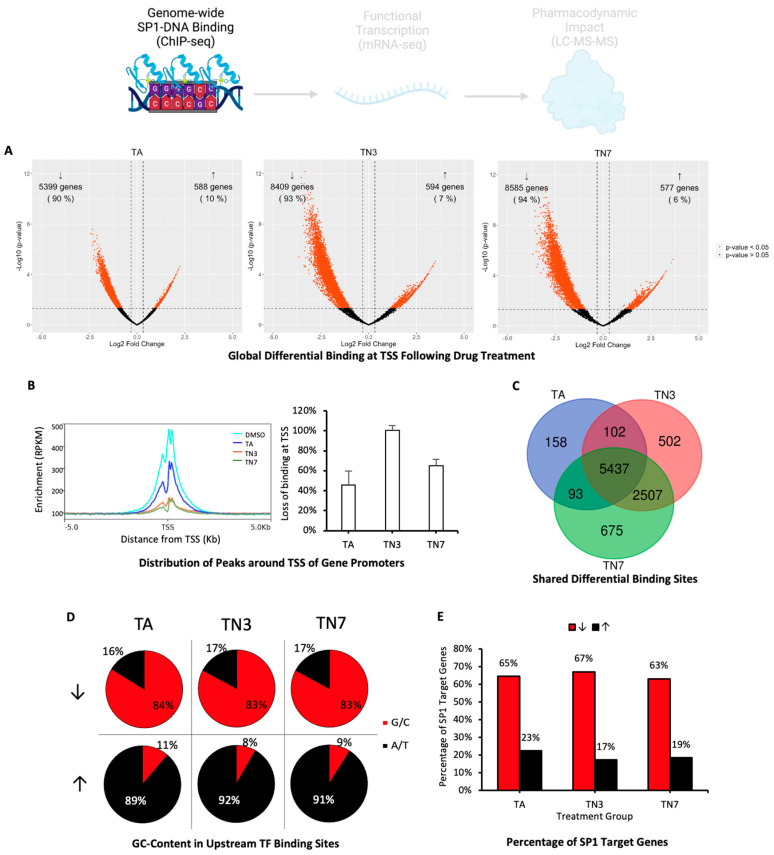
Genome-wide differential SP1-DNA binding analysis (ChIP-Seq). Differentiated SH-SY5Y cells were primed with lead acetate for 48 h and subsequently treated with 25 µM of tolfenamic acid (TA), TN3, or TN7 for 72 h. Differential binding analysis was conducted using ChIP-Seq. (**A**) Volcano plots demonstrating binding sites up to 500 bp upstream of transcription start sites (TSSs) exhibiting reduced (↓) or enhanced (↑) SP1-DNA binding following treatment with TA, TN3, or TN7. (**B**) Distribution and quantification of ChIP-seq peaks (reads per kilobase million) around TSSs (−/+5 kb) across the genome after treatment with TA, TN3, or TN7. (**C**) Venn diagram showing the number of genes affected in each treatment group and overlapping genes. (**D**) Pie chart representing the average percentage of G/C and A/T content in transcription factor (TF) binding sites found upstream of the TSSs of genes with reduced (↓) or enhanced (↑) SP1-DNA binding at their promoter sites. (**E**) Bar chart representing the percentage of SP1 targets within the genes with reduced (↓) or enhanced (↑) SP1-DNA binding upstream of their TSSs.

To examine the genome-wide effect of drug treatment on SP1-DNA binding, ChIP-Seq analysis was performed using the SP1 antibody in differentiated and lead-induced SH-SY5Y cells treated with TA, TN3, or TN7 and compared with the control (Figure 3). The analysis of differential binding occurring up to 500 bp upstream of transcription start sites (TSSs) revealed a high percentage of decreased SP1-DNA binding in all treatment groups (TA: 5399 genes (90%); TN3: 8409 genes (93%); TN7: 8585 genes (94%)), while increased SP1-DNA binding occurred only in 6–10% of the affected genes (Figure 3A). The analysis of genome-wide average read density around TSSs demonstrated that drug treatment reduced SP1-DNA binding at regions closest to TSSs, with TA being the least potent and TN7 being the most potent (Figure 3B). A Venn diagram of the number of genes exhibiting significant differential binding at their promoter regions showed that 5437 (57%) of all affected genes were common between all groups, representing 94% of the genes affected by TA (Figure 3C). TN3 and TN7 also exhibited a high degree of overlap, with 7944 (93%) of the genes affected by TN3 treatment also present in the TN7 group (Figure 3C). Promoter analysis revealed that GC-rich (TA: 84%; TN3: 83%; TN7: 83% G/C) transcription factor (TF) binding sites, mostly zinc finger-type sites and including the SP1 binding site (Table S1), were enriched in sites where SP1 exhibited decreased binding following drug treatment (Figure 3D). In contrast, AT-rich (TA: 89%; TN3: 92%; TN7: 91% A/T) TF-binding sites were enriched in sites where increased SP1-DNA binding occurred (Figure 3D), including for TATA-binding proteins (TBP) (Table S1). Furthermore, a significant proportion of the binding sites exhibiting decreased binding of SP1 following drug treatment belonged to genes that are known to be downstream targets of SP1 (TA: 65%; TN3: 67%; TN7: 63%) (Figure 3E), while SP1 targets represented only a minimal proportion of sites that exhibited increased SP1 binding (TA: 23%; TN3: 17%; TN7: 19%) (Figure 3E).

### 2.4. Transcriptomic Analysis (mRNA-Seq) Revealed Differential Expression of SP1-Driven Gene Expression

**Figure 4:**
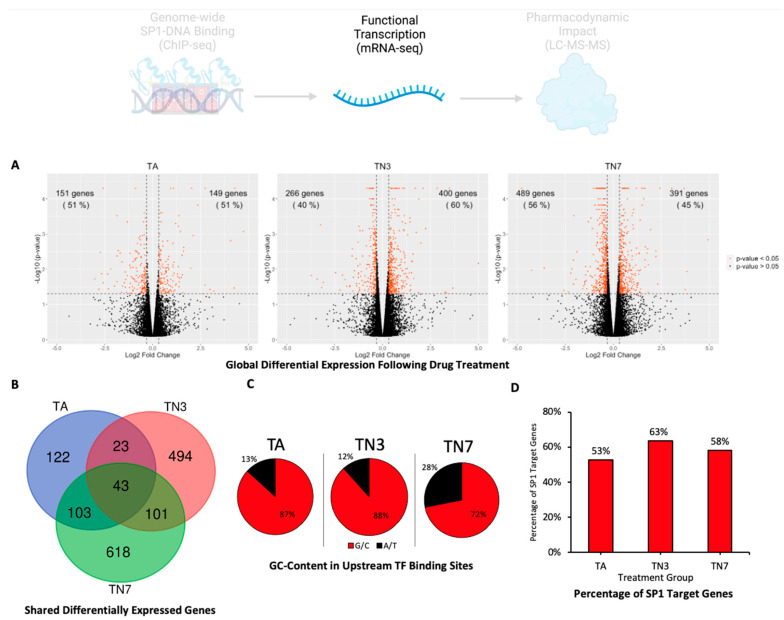
Differential transcriptomic expression analysis (mRNA-seq). Differentiated SH-SY5Y cells were primed with lead acetate for 48 h and subsequently treated with 25 µM of TA, TN3, or TN7 for 72 h. Differential expression analysis was conducted using mRNA-seq. (**A**) Volcano plots demonstrating differentially expressed mRNAs following treatment with TA, TN3, or TN7. (**B**) Venn diagram showing the number of mRNAs perturbed in each treatment group and overlapping mRNAs. (**C**) Pie chart representing the average percentage of G/C and A/T content in transcription factor (TF) binding sites in the promoter region 300 bp and 600 bp upstream of the differentially expressed genes. (**D**) Bar chart representing the percentage of SP1 targets within the differentially expressed genes.

To assess the functional consequences of differential SP1 binding to multiple DNA sites, we performed transcriptomic analysis on cells treated with TA, TN3, or TN7 (Figure 4A). A total of 291 mRNAs were differentially expressed following TA treatment, 58% of which (169 mRNAs) were common with either TN3- or TN7-treated groups (Figure 4B). Furthermore, 144 (22%) of the mRNAs differentially expressed following treatment with TN3 were also perturbed by TN7 treatment (Figure 4B). GC-rich (TA: 87%; TN3: 88%; TN7: 72% G/C) TF-binding sites were found to be enriched in the regions upstream of the TSSs of the differentially expressed genes (Figure 4C and Table S2). SP1 targets represented a considerable fraction of these genes (TA: 53%; TN3: 63%; TN7: 58%) (Figure 4D).

### 2.5. TA, TN3, and TN7 Lower AD-Related Proteins and Modulate the Global Proteome

**Figure 5:**
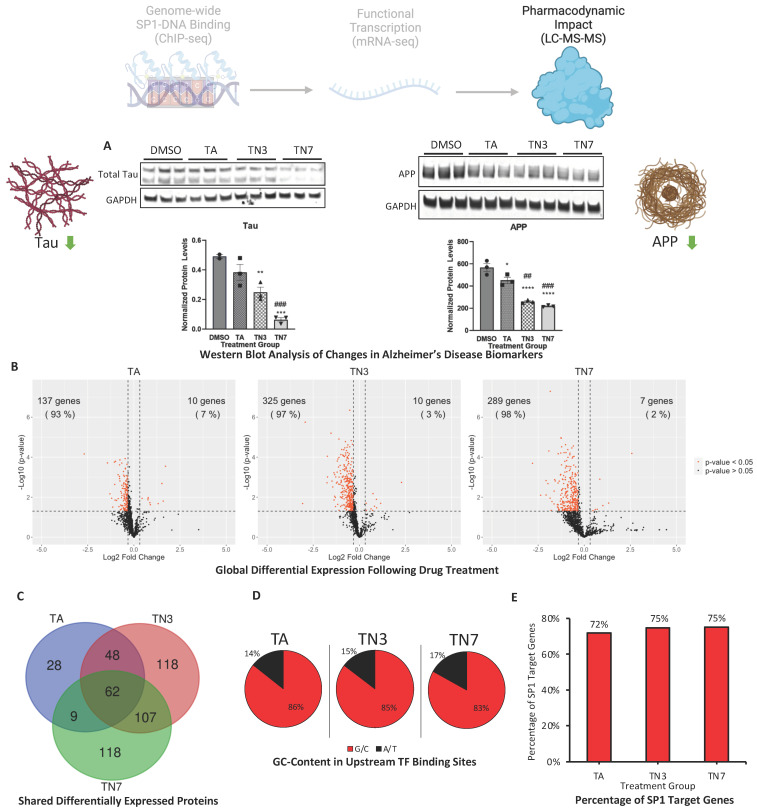
Proteomic differential expression analysis by SWATH LC–MS-MS DIA. Differentiated SH-SY5Y cells were primed with lead acetate for 48 h and subsequently treated with 25 µM TA, TN3, or TN7 for 72 h: (**A**) Western blot analysis was performed on total protein lysates. Quantification normalized to GAPDH is shown. Data are expressed as ± S.E.M. Statistical significance was determined using one-way ANOVA followed by Dunnett’s test for multiple comparisons, and n was 3 for each treatment group. (* *p* < 0.05, **/## *p* < 0.01, ***/### *p* < 0.001, and **** *p* < 0.0001); * denotes comparison to DMSO control, and # denotes comparison to TA. Global proteomic analysis was conducted using SWATH-LC–MS/MS DIA. (**B**) Volcano plots demonstrating differentially expressed proteins following treatment with TA, TN3, or TN7. (**C**) Venn diagram showing the number of proteins perturbed in each treatment group and overlapping proteins. (**D**) Pie chart representing the average G/C and A/T content (%) in transcription factor (TF) binding sites in promoter regions 300 bp and 600 bp upstream of the differentially expressed genes. (**E**) Bar chart representing the percentage of SP1 targets within the differentially expressed genes.

Western blot analysis revealed that TN3 and TN7 significantly lowered the total Tau protein levels, while TA had a lesser effect (Figure 5A). Furthermore, TN3 and TN7 outperformed TA in lowering APP protein levels (Figure 5A). To determine the global downstream functional effect of each drug treatment, proteomic analysis using liquid chromatography with tandem mass spectrometry (LC-MS-MS) was carried out. Volcano plots demonstrated that treatment with TA, TN3, or TN7 downregulated more proteins than those that were upregulated (Figure 5B). The Venn diagram demonstrated that approximately 75% of perturbed proteins in the TA treatment group were common with TN3 (Figure 5C). Additionally, 57% of differentially expressed proteins in TN7 were shared with the TN3 group (Figure 5C). The promoter analysis of the genes of differentially expressed proteins showed enrichment of upstream GC-rich (TA: 86%; TN3: 85%; TN7: 83% G/C) TF-binding sites (Figure 5D and Table S2). Interestingly, SP1 targets represented the great majority of the differentially expressed proteins revealed using LC-MS-MS (TA: 72%; TN3: 75%; TN7: 75%) (Figure 5E).

### 2.6. TN3 and TN7 Are More Potent Inhibitors of Neuronal Pathways

**Figure 6:**
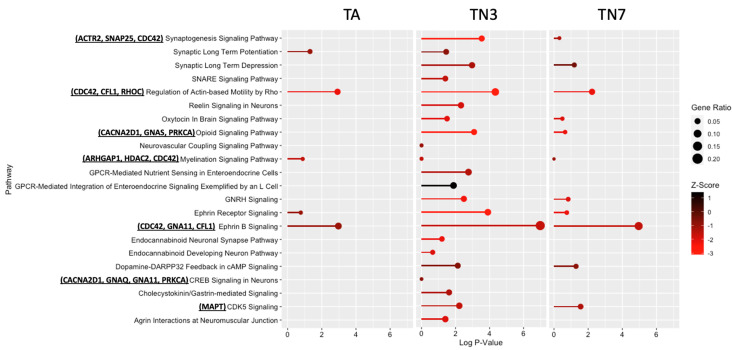
Neuronal pathways affected by treatment with TA, TN3, or TN7: Global proteomic analysis was conducted using SWATH-LC-MS/MS DIA. The data were analyzed using Spectronaut, Qiagen IPA, and R. The values are based on the proteomic analysis of each treatment group compared with the control group filtered for neurological pathways.

Functional enrichment analysis was carried out to assess the main pathways related to neurotransmitters and other types of nervous system signaling that are enriched in each treatment group using Qiagen’s IPA platform [20] (Figure 6). The analysis of the differentially perturbed proteins following treatment with TA, TN3, and TN7 demonstrated that treatment with TN3 or TN7 likely inhibited more pathways than treatment with TA, with TN3 being the most potent inhibitor both in terms of the number of proteins perturbed and the extent of perturbation within individual pathways (Figure 6 and Table S3). The impacted neuronal pathways include those involved in synaptic development, neuronal connectivity, actin-based motility, synaptic plasticity, neuroprotection, and myelination. Additionally, the pathways and proteins affected by TA were similarly targeted by TN3 and TN7, and the pathways and proteins impacted in the TN7 group exhibited overlapping perturbations with those in the TN3 group (Figure 6 and Table S3). Finally, the functional categorization of the perturbed proteins revealed associations with key biological functions encompassing cytoskeleton and actin regulation, gene regulation, protein phosphorylation, and vesicle transport (Table 1).

## 3. Discussion

Small-molecule approaches for the treatment of AD and other neurogenerative diseases should not be overlooked. Our approach combines the ease and great potential of small molecules with genomic targeting that can offer alternatives to biologics and nucleic acid-based therapies. We aimed to employ a multiomic approach to profile the extent of the effects of mechanism-based small molecules that acted upstream to impact AD-related genes and their regulators. We investigated the genome-wide effect on SP1-DNA binding and the global differentially expressed transcriptomic and proteomic profiles in differentiated SH-SY5Y cells treated with TA and its analogs (TN3 or TN7). While these cells are of dopaminergic neuronal lineage, they are considered a well-established model for neurotoxicity and neuroprotection studies, including those related to AD. The selection of SH-SY5Y cells was based on several considerations. These include their human neuronal origin, which aligns with the objective of mimicking human neuronal responses. While they do not perfectly replicate primary neurons, it is important to note that SH-SY5Y cells retain key neuronal properties, such as the capacity for differentiation and responsiveness to neurotrophic factors and neurotoxic stimuli. 

The results of this study suggest that the drugs TA, TN3, and TN7 have a common SP1-mediated mode of action. First, we performed EMSA analysis, which confirmed that each small molecule prevented SP1-DNA binding to its consensus element, which is consistent with earlier reports for TA in non-neural tissue [21] (Figure 2B). Furthermore, the genome-wide loss of SP1-DNA binding revealed using ChIP-Seq suggests that SP1 plays a primary role in the drugs’ downstream effects (Figure 2B and Figure 3A). The high similarity in the genes affected between treatment groups further supports a similar mode of action, with TA displaying the least effect compared with TN3 and TN7. Expectedly, the genomic regions that underwent SP1-DNA binding loss (63–67%) were known SP1 targets (Figure 3E). Only a minority of the genes exhibited increased SP1-DNA binding (6–10%). These were not known to be SP1 targets and lacked the canonical GC-rich SP1-binding elements in their promoters (Figure 3D,E and Table S1). This may be explained by the consequent increased availability of SP1 and, thus, an increase in SP1 protein interactions with cofactors that bind to AT-rich motifs such as TATA-binding proteins (TBPs) [22] (Table S1). 

DNA binding does not necessarily mean transcriptional activity. Thus, we observed a remarkable decline in the number of perturbations as we moved downstream from differential binding using ChIP-Seq, which showed a high number of events (9474 genes), to mRNA-Seq, which revealed a smaller number of changes (1504 genes); and finally, LC-MS-MS, which detected the lowest number of changes at the proteomic level (total of 490 proteins). This difference is favorable and anticipated since many alterations in binding at the genome level may not result in transcriptional changes [22,23,24,25]. Similarly, not all transcripts are translated into proteins, and some transcripts may have regulatory or structural roles that do not affect the proteome [26].

The transcriptomic and proteomic profiles of SH-SY5Y cells treated with TA, TN3, and TN7 revealed using mRNA-seq and LC-MS-MS further agreed to a SP1-mediated mode of action (Figure 4 and Figure 5). Treatment with TA, TN3, or TN7 resulted in a proteomic shift compared to the control with overlapping proteomic changes (Figure 5C). Since TN3 and TN7 shared the greatest number of differentially expressed proteins compared to their overlaps with TA, this may be indicative of the similar pharmacodynamics of TN3 and TN7 (Figure 5C). Similar proteomic profiles were expected as TN3 and TN7 were synthesized using TA as a scaffold structure (Figure 1B). Volcano plot analysis of the perturbed proteins revealed that TA, TN3, and TN7 were likely upstream inhibitors as they downregulated more pathways than they upregulated (Figure 5B). The perturbed proteins were products of genes with GC-rich TF-binding motifs (83–86% G/C), with SP1 targets representing the majority (72–75%) of these proteins (Figure 5D,E). Taken together, these results support the SP1-specific mode of action for TA and its analogs (Figure 5C). 

Interestingly, TN3 and TN7 outperformed TA in lowering the protein expression of the AD-related biomarkers, Tau and APP, which are SP1 target genes (Figure 5A). It is worth emphasizing that Tau is not exclusive to AD pathology and is implicated in various neuropathological conditions (e.g., frontotemporal dementia, progressive supranuclear palsy, amyotrophic lateral sclerosis, etc.). Nevertheless, this outcome signifies the drugs’ capability to decrease both Tau and APP proteins, which is not achieved with the currently approved AD therapies. Drugs in development for AD may target either Tau or APP but not both [27]. For example, in a recent phase 1b trial, a Tau-targeting antisense oligonucleotide (MAPTRx) was found to reduce Tau mRNA and protein by 50–80% in patients with mild AD [28]. Notably, this trial also reported adverse events in 94% of drug-treated patients and 75% of placebo-treated patients, mostly mild or moderate headaches after injection [28]. In another study, engineered zinc finger protein transcription factors (ZFP-TFs) were used to repress Tau expression in the brain of mice, achieving a similar degree of Tau reduction [29]. However, neither of these drugs affected APP levels or Aβ pathology. In contrast, our compounds can simultaneously lower both Tau and APP proteins, which may have synergistic effects on the prevention or reversal of AD-related neurodegeneration. 

To investigate the neuronal pathways that may be affected by each treatment, we examined the proteomic impact of treatment with TA, TN3, or TN7, and we found that these drugs would decrease several pathways of interest (Figure 6). Interestingly, these pathways are also affected by NMDA receptor agonists and antagonists, which may indicate that this is an upstream target [30,31,32]. Notably, CDK5 signaling was also predicted to be decreased by TN3 and TN7, with MAPT being one of the genes affected, which further validates the proposed mechanism of action of SP1-DNA binding inhibition (Figure 1A). 

Overall, the impact on common neuronal pathways was observed, suggesting a common mechanism of action, with TN3 demonstrating the most significant effects, whereas TA exhibited the least in terms of both the number of proteins affected within the pathway and the extent of perturbation (Figure 6 and Table S3). The treatments impacted the proteins involved in crucial biological processes, including cytoskeleton regulation, synaptic plasticity, and neurotransmission. They also affected signaling pathways, G proteins, GTP-binding proteins, calcium channel regulation, and vesicle transport, all essential for neuronal communication and function. These findings suggest the potential mechanisms through which these drugs influence AD-related processes, emphasizing their focused nature and the need for further investigations.

## 4. Materials and Methods

### 4.1. Design and Synthesis of Tolfenamic Acid (TA) Analogs

We focused our design of TA derivatives on potency and selectivity according to the Lipinski rule to optimize molecular weight and properties for blood–brain barrier (BBB) penetration, such as lipophilicity, molecular weight, and the number of hydrogen bond donors and acceptors. Using the structure–activity relationship, four modifiable regions in TA were identified (Figure 1B). Initial molecular orbital calculations with complete geometry optimizations were performed at the Hartree–Fock level utilizing a 6–31G* basis set for TA and the proposed derivatives. The resulting conformations after modifications were superimposed on a prototype TA conformation to determine the effect of functional group modification on the conformation of the parent compound TA. Next, the tendency of each compound to bind zinc ions in the gas phase was studied. Complete geometry optimizations were carried out in the presence of zinc, allowing the compound to reorganize to capture the ion in the most efficient way. The overall binding affinity was then viewed as a combination of stabilization gained in binding and the loss in conformational rearrangement required for the efficient capture of the ion. Interaction energy or stabilization in binding, as well as conformational reorganization, were calculated as follows: interaction energy = E_complex_ − (E_zinc_ + E_drug_); conformational reorganization = (E_reorganized drug_ − E_drug before ion binding_). The ionizable groups in the drug were used in ionized form. All calculations were carried out using Gaussian ‘09 software, and Gaussview was used for viewing purposes (data available). 

Based on the modeling studies, group modifications that demonstrated better interaction energy and conformation reorganization when compared with TA (interaction energy = −419.32 kcal/mol; reorganization energy = 8.13 kcal/mol) were further analyzed. To understand the effects on the inhibition of the SP1-DNA binding, a model of zinc finger protein SP1 (PDB ID: 1VA1 corresponding to zinc finger 1 of SP1 [19]) was prepared for docking studies; that is, the hydrogen atoms were added, and the catalytic motif C2H2 was identified. All the residues ionized at physiological pH were changed to their ionized forms. TA and its derivatives were then manually docked one by one near the Zn ion and the catalytic motif (Figure 2A). The interaction between the zinc finger and the drug was then calculated. To calculate the interaction energy, the energy of the prepared zinc finger protein was calculated at the 3–21G level. Single-point calculations were also carried out for the prepared drug molecules at the 3–21G basis set. The compound was then docked in and the energy of the complex supermolecule was calculated. Interaction energy was calculated as follows: interaction energy = E_complex supermolecule_ − (E_drug_ + E_zinc finger_). Based on the modeling studies with Zn^2+^ and SP1, we selected rationally specific compounds in these four regions and eliminated a number of compounds (data available). 

Synthetic routes using appropriately substituted benzoic acid and aniline derivatives, as well as other substituted aromatic or heteroaromatic compounds, were employed. For example, benzoic acid with suitable leaving groups (e.g., -Br) was reacted with substituted aniline derivatives to generate compounds with diverse substituents in region “III”. The esterification of TA derivatives with corresponding alcohols using 2,2-dicyclohexylcarbodiimide (DCC) and catalytic amounts of 4-dimethylaminopyridine yielded ester derivatives in the region “I”. Similarly, ether and thiol derivatives in group “II” were prepared by utilizing phenol or thiophenol derivatives instead of aniline derivatives. The selected compounds were subsequently screened using cell viability assays (Figure 1B). 

### 4.2. Cell Culture

Human neuroblastoma SH-SY5Y cells were purchased from the American Type Culture Collection (Cat. CRL-2266, ATCC, Manassas, VA, USA). Cells were cultured in Dulbecco’s modified Eagle medium (DMEM)/F12 with the GlutaMAX supplement (Cat. 10565018, ThermoFisher Scientific, Waltham, MA, USA) supplemented with 10% heat-inactivated fetal bovine serum (FBS) (Cat. 16140071, ThermoFisher Scientific, Waltham, MA, USA) and 1% antibiotic–antimycotic (Cat. 15240062, Gibco, ThermoFisher Scientific, Waltham, MA, USA) in T-75 tissue culture flasks with vented lids (Cat. CC7682-4875, USA Scientific Inc., Ocala, FL, USA) in a CO_2_ incubator maintained at 5% CO_2_ and 37 °C. Cells were then subcultured at a density of 10^5^ cells/mL, and upon 70% confluency, the cells were differentiated for 6 days using 10 µM all-trans retinoic acid (Cat. R2625, Sigma-Aldrich, St. Louis, MO, USA) in Neurobasal medium (Cat. 21103049, ThermoFisher Scientific, Waltham, MA, USA) supplemented with 1% serum-free B-27 (50X) (Cat. 17504044, ThermoFisher Scientific, Waltham, MA, USA) in the dark. Cells were observed for neurite out-growth, with media being changed every 48 h as described previously [33]. Furthermore, the differentiated cells were exposed to 25 µM of lead acetate trihydrate (Cat. 215902, Sigma-Aldrich, St. Louis, MO, USA) for 48 h to induce AD biomarkers. Afterward, cells were washed with 1X PBS and then treated with tolfenamic acid (Cat. T0535, Sigma-Aldrich, St. Louis, MO, USA), TN3, or TN7 at 25 µM dissolved in DMSO and incubated for 72 h. 

### 4.3. Cell Viability Assay

The viability of differentiated SH-SY5Y human neuroblastoma cells was assessed after exposure to TA, TN3, and TN7 using a Vybrant^TM^ MTT Cell Proliferation Assay Kit (Cat. V13154, Invitrogen, Leiden, The Netherlands). Cells were seeded in a 96-well plate (2 × 10^4^ cells/well) and differentiated as described above. The cells were then treated with different concentrations of TA, TN3, and TN7 as specified in the differentiation medium for 72 h. After the completion of the incubation period, the medium was aspirated out and 100 µL of a fresh, phenol-red-free medium was added to each well, followed by treatment with 10 µL of the MTT solution. The plates were incubated at 37 °C for 4–18 h, and after incubation, the absorbance was measured at 570 nm using a microplate reader (TECAN). 

Compounds of interest with superior safety profiles, TN3 (Cat. 225038, Santa Cruz Biotechnology, Dallas, TX, USA) and TN7 (Cat. 4112, TOCRIS, Minnesota, MN, USA), were obtained from commercial sources for further testing. 

### 4.4. Electrophoretic Mobility Shift Assay (EMSA)

The cells were washed using 1X PBS and then lysed using 1X radio-immunoprecipitation assay (RIPA) lysis buffer (Cat. 9806S, Cell Signaling Technology, Danvers, MA, USA) supplemented with a phosphatase and protease inhibitor tablet (Cat. A32961, ThermoFisher Scientific, Waltham, MA, USA). The cells were subsequently incubated on ice for 5 min, scaped into a microcentrifuge tube, sonicated twice for 15 s using a VWR Ultrasonic Homogenizer (Cat. 79193-588, VWR International, Radnor, PA, USA), and centrifuged at 14,000× *g* for 10 min, and then the supernatants were collected and stored at −80 °C for protein quantification and Western blot analysis. The protein concentration was determined using a Micro BCA Protein Assay Kit (Cat. 23235, ThermoFisher Scientific, Waltham, MA, USA) according to the manufacturer’s protocol. 

Biotinylated and unbiotinylated DNA oligonucleotides for the SP1 consensus sequence (5′-GCTCGCCCCGCCCCGATCGAAT-3′) and the complement were purchased from Integrated DNA Technologies (IDT, Coralville, IA, USA). Oligos were dissolved in nuclease-free water to obtain a concentration of 100 µM. Then, the oligos were annealed by first diluting each to 5 µM in nuclease-free water and then heating them to 90–95 °C in a heat block and subsequently placing them in a water bath to allow them to be slowly cooled back to room temperature. The annealed oligos were stored at −20 °C. Binding reactions were carried out using a LightShift Chemiluminescent EMSA Kit (Cat. 20148, ThermoFisher Scientific, Waltham, MA, USA). The binding reactions were carried out according to the manufacturer’s protocol using a 1X binding buffer, 50 ng/µL of poly(dI/dC or poly AT), 4 µg of total protein from the treated cells described above, 20 fmol of labeled or 4 pmol unlabeled target DNA, and ultrapure water in a 20 µL total reaction volume. EDTA was used as a positive control. The reaction was performed while incubating on ice for 15–30 min. Electrophoresis was carried out with 6% non-denaturing acrylamide gels using 29:1 acrylamide:bis-acrylamide. The gels were run at 100 V at room temperature. Samples were transferred onto BrightStar Plus Positively Charged Nylon Membranes (Cat. AM10100, ThermoFisher Scientific, Waltham, MA, USA). DNA was cross-linked to the membrane for 1 min at 120 mJ/cm^2^. DNA was detected using the Pierce Chemiluminescent Nucleic Acid Detection Module supplied in the kit according to the manufacturer’s protocol. Images were captured using a C-DiGit Blot Scanner (LI-COR, Lincoln, NE, USA). 

### 4.5. Chromatin Immunoprecipitation Sequencing (ChIP-Seq) Sample Preparation

Cells were prepared for ChIP reactions, and all reactions were conducted using a SimpleChIP Plus Chromatin Immunoprecipitation Kit (Cat.9005, Cell Signaling Technology, Danvers, MA, USA) according to the manufacturer’s protocol. Briefly, the cells were cross-linked using 37% formaldehyde (Cat. F8775, Sigma-Aldrich, St. Louis, MO, USA) at a 1% final concentration in 20 mL of complete media for 1 min at room temperature. Then, 2 mL of 10X glycine was added to each flask of cells and incubated at room temperature for 5 min. The medium was removed, and the cells were washed twice with 1X PBS. Then, the cells were scraped using ice-cold PBS supplemented with the protease inhibitor included in the kit. Afterward, cells were centrifuged at 2000× *g* for 5 min at 4 °C. Nuclei preparation was conducted using the manufacturer’s protocol, with minor modifications. Nuclei were digested using 0.5 µL Micrococcal Nuclease per 4 × 10^6^ cells at 37 °C, and the tube was inverted every 3 min for 20 min before the reaction was stopped using EDTA. The reaction was pelleted via centrifugation at 16,000× *g* for 1 min at 4 °C, the supernatant was transferred to a new tube, and the lysate was sonicated 3 times for 15 s and allowed to rest 2–3 min between each sonication using the VWR Ultrasonic Homogenizer (Cat. 79193-588, VWR International, Radnor, PA, USA). Afterward, the lysates were clarified via centrifugation at 9400× *g* for 10 min at 4 °C. Chromatin digestion was analyzed using a 1% agarose gel to electrophorese the DNA with a 100 bp DNA marker. DNA was digested to be between 150 and 900 bp. DNA concentration was determined using the manufacturer’s recommendations. Chromatin immunoprecipitation reactions were carried out overnight (15–20 h) at 4 °C in rotation using 5–10 µg of digested chromatin, a ChIP buffer supplemented with a protease inhibitor cocktail, and 1.3–2.6 µg of SP1 ChIP-validated Rabbit mAb antibody (Cat. 9389, Cell Signaling Technology, Danvers, MA, USA) or 1.3–2.6 µg of normal Rabbit IgG (Cat. 2729S, Cell Signaling Technologies, Danvers, MA, USA) which was the negative control. Histone H3 XP Rabbit mAB was used as a positive control. ChIP-grade protein G beads were added to each reaction and incubated for 3 h at 4 °C with rotation. Protein G beads were pelleted using MagneSphere Technology Magnetic Separation Stands (Cat. Z5341, Promega, Madison, WI, USA) and washed three times with a low salt wash and once with a high salt wash, pelleting the chromatin and removing the supernatant after each wash, per the manufacturer’s instructions. To elute the DNA, the magnetic beads were suspended in 1X ChIP elution buffer, and the samples were incubated at 65 °C and 1200 rpm for 30 min in the Eppendorf ThermoMixer C (Cat. 5382000023, Eppendorf, Enfield, CT, USA). Following elution, the magnetic beads were pelleted, the supernatant containing chromatin was transferred to a new tube, and the cross-links were reversed via incubation at 65 °C for 2 h with proteinase k. The samples were purified using the supplied DNA purification kit according to the manufacturer’s protocol. 

The relative enrichment of the target regions in the precipitated DNA fragments was first analyzed via qPCR (Applied Biosystems, Foster City, CA, USA) using Fast SYBR Green Master Mix (Applied Biosystems, Foster City, CA, USA) for the quality control of the ChIP. Once the ChIP QC was assessed, the DNA library preparation was performed using a NEXTflex ChIP-Seq Barcodes 6 Kit (Bioo Scientific, Austin, TX, USA), and then the quality of the library generated was assessed on an Agilent 2100 Bioanalyzer system followed by accurate quantification using the Qubit system. The libraries that passed quality control were pooled, clustered on a cBot platform, and sequenced on an Illumina HiSeq 4000 system at a minimum of 25 million paired-end reads (2 × 75 bp) per sample.

### 4.6. Total RNA Isolation for Transcriptomic Analysis

The total RNA was isolated from the cells using the TRIzol reagent method (Cat. 15596018, ThermoFisher Scientific, Waltham, MA, USA) according to the manufacturer’s protocol. The purity and concentration of the isolated RNA were verified using a NanoDrop One Microvolume UV–Vis Spectrophotometer (Cat. ND-ONEC-W, ThermoFisher Scientific, Waltham, MA, USA). First-strand complementary DNA (cDNA) was synthesized from 0.5 μg of total RNA using an iScript cDNA Synthesis Kit (Cat. 1708891, Bio-Rad, Hercules, CA, USA). Briefly, the total RNA with a RIN number above 8 was used as input for library preparation using a TruSeq Stranded mRNA Kit (Cat #: 20020594) from Illumina, following the manufacturer’s protocol. Briefly, from 500 ng of total RNA, mRNA molecules were purified using poly-T oligo-attached magnetic beads, and then mRNA was fragmented. cDNA was generated from the cleaved RNA fragments using random priming during first- and second-strand syntheses. Barcoded DNA adapters were ligated to both ends of the DNA and then amplified. The quality of the library generated was assessed on an Agilent 2100 bioanalyzer system and quantified using the Qubit system. Libraries that passed quality control were pooled, clustered on a cBot platform, and sequenced on an Illumina HiSeq 4000 system at a minimum of 20 million paired-end reads (2 × 75 bp) per sample.

### 4.7. Protein Isolation for Proteomic Analysis

Briefly, 8.4 × 10^6^ cells were collected into a 15 mL conical tube using a cell scraper. Then, the tubes were centrifuged at 1500 rpm for 3 min at RT, the supernatant was discarded, and the cells were washed 3× using 1× PBS. Following the final wash, the cells were resuspended in 1 mL of homogenization buffer (8M Urea, 50 mM triethylammonium bicarbonate (TEAB), and 10 mM DTT in sterile water). Lysate homogenization, BCA analysis, and relative protein quantification with SWATH-MS (sequential window acquisition of all theoretical mass spectra) were performed as previously described [34], with some modifications. Briefly, protein samples (250 µg) were spiked with 2 µg of BSA and denatured with 25 µL of DTT (100 mM) at 37 °C for 15 min in a shaking water bath (100 rpm). After denaturation, the lysates were alkylated in the dark with 25 µL IAA (200 mM) for 30 min at room temperature. The protein was precipitated using 250 µL of ice-cold methanol and vortexed. Then, the samples were vortexed again following the addition of 250 µL of chloroform. To complete precipitation, the lysates were centrifuged at 12,000 rpm, 5 min at 10 °C. The protein pellet was washed with ice-cold methanol and then gently resuspended in 130 µL of 50 mM ammonium bicarbonate (pH 8) containing 3% *w/v* sodium deoxycholate (DOC). TPCK-treated trypsin was added to the samples at a ratio of 1:20 (trypsin–protein), and they were then transferred into digestion tubes (PCT MicroTubes, Pressure Biosciences Inc., Easton, MA, USA). The digestion procedure was carried out using a barocycler at 37 °C, for 75 cycles with 60 s per pressure-cycled (50 s high pressure and 10 s ambient pressure, at 25.5 kpsi). A second digestion procedure was performed by adding the same amount of fresh trypsin for an additional 60 cycles. Furthermore, 135 µL of the digested peptide sample was mixed with 15 µL of ACN (1:1, *v/v* containing 5% formic acid) to precipitate the detergent. Samples were centrifuged to remove the pellet, and 100 µL of the supernatant was collected (12,000 rpm for 5 min at 10 °C). Subsequently, 90 µL of the peptide solution was injected into the analytical column and was analyzed using LC-MS/MS. Data-independent acquisition was carried out on a SCIEX 5600 TripleTOF mass spectrometer (SCIEX, Concord, ON, Canada) coupled to the Acquity UHPLC H-class liquid chromatography system (Waters Corp., Milford, MA, USA). Peptide separation was achieved using a run time of 60 min at 100 μL/min and a linear gradient method. Spectronaut™ was used for peptide identification and data extraction from SWATH files. 

### 4.8. Western Blotting

For Western blotting, 15–20 µg of total protein was resolved using 4–12% Bolt Bis-Tris pre-cast gels (Cat. NW04125BOX, ThermoFisher Scientific, Waltham, MA, USA) and Bolt MES SDS running buffer (Cat. B0002, ThermoFisher Scientific, Waltham, MA, USA) at 200 V constant under reducing conditions at room temperature according to the manufacturer’s protocol. The protein was transferred onto 0.4 µm polyvinylidene fluoride (PVDF) membranes (GE-Healthcare, Cleveland, OH, USA) using cold 1× Bolt transfer buffer (Cat. BT0006, ThermoFisher Scientific, Waltham, MA, USA) at 4 °C for 2.5 h. The membranes were blocked for 1 h in Sea Block Blocking Buffer (Cat. 37527x3, ThermoFisher Scientific, Waltham, MA, USA) and incubated overnight with primary antibodies (Tau: Tau46; APP: (E8B30) XP; GAPDH: (D16H11) XP, CST, MA, USA) with gentle agitation on a shaker at 4 °C. The antibodies were detected using InfraRed detectable secondary antibodies (Cat. 926-68070 or 926-68071 LI-COR Biotechnology, Lincoln, NE, USA), and imaged using a LI-COR Odyssey CLX infrared scanner. Band intensities were measured using Image Studio Lite v5.0 and normalized against GAPDH.

### 4.9. Promoter Analysis

To identify the enriched transcription factor binding motifs in the promoters of the affected genes, we utilized the Integrated Differential Expression and Pathway (iDEP) analysis tool. Essentially, sequences located 300 bp and 600 bp upstream of the genes of interest were analyzed.

SP1 general targets retrieved from the Open Source Database for Human Transcription Factor Targets (http://bioinfo.life.hust.edu.cn/hTFtarget#! (accessed on 23 May 2023)) were used to quantify the percentage of SP1 targets within the datasets [35].

### 4.10. Data Analysis and Visualization

The raw data for the volcano plots, Venn diagrams, and heatmaps were analyzed and visualized in R v2023.03.1+446 using Bioconductor v3.16, Limma v3.52.4, GGplot2 v3.4.0, and VennDiagram v1.7.3 [36,37,38,39]. 

For pathway analysis, the pathways were identified from the QIAGEN Ingenuity Pathway Analysis Library of canonical pathways associated with neurotransmitters and other types of nervous system signaling that were most significant to the dataset. The molecules from the dataset that met the fold change cutoff of 1.25 and a *p*-value cutoff of 0.05 and were associated with a canonical pathway in the QIAGEN Knowledge Base were considered for the analysis. The significance of the association between the dataset and the canonical pathway was measured as follows: (1) gene ratio: a ratio of the number of molecules from the dataset that map to the pathway divided by the total number of molecules that map to the canonical pathway is displayed; (2) log *p*-value: a right-tailed Fisher’s exact test was used to calculate the *p*-value determining the probability that the association between the genes in the dataset and the canonical pathway explained by chance alone; and (3) z-score: a z-score was calculated to indicate the likelihood of the activation or inhibition of that pathway. The visualization of the top neuronal pathways was carried out in R v2023.03.1+446.

## 5. Conclusions

We used this mechanism-based approach to develop safer and more potent derivatives of TA with proven impacts on AD-related pathways. In summary, we proposed a small-molecule approach that can act at the gene level and specifically modulate the transcription of AD-related genes with limited systemic and off-target effects. While we utilized SH-SY5Y cells for our in vitro experiments, chosen for their relevance and established use in neurodegenerative research, we acknowledge their limitations. To address these concerns, in vivo studies in mouse models of AD are ongoing, offering a more comprehensive view of our compounds’ potential. We present this method as a promising approach for developing affordable, accessible, and safe drugs for the treatment of AD.

## 6. Patents

TN3 (63/365,349, patent pending) and TN7 (63/396,855, patent pending) may be under US Patent and Trademark Office (USPTO) patent protection if the patents are approved by the time of publication.

## Figures and Tables

**Table 1:** Proteins within neuronal pathways affected by treatment with TA, TN3, and TN7 grouped into functional categories.

Functional Category	Proteins
**Cytoskeleton and actin regulation**	ACTA1, ACTR2, ARPC2, ARPC3, CDC42, CFL1, CTNNB1, CAP1, GNA11, GNAI3, GNAQ, GNAS, GNG2, RAP1B, RAB1A, RAB2A, RHOC
**Gene regulation**	NAP1L1, NAP1L4, SMARCC2, HDAC2, LMN1B, HNRNPK
**Phosphatases/kinases**	ACP1, PPP2CB, PPP2R1A, PPP3CA, PRKCA
**Vesicle transport**	AP2A2, AP2B1, AP2M1, SNAP25

## Data Availability

Data are contained within the article or supplementary material.

## Supplementary Materials

The following supporting information can be downloaded at: https://www.mdpi.com/article/10.3390/ijms242015216/s1.
