# Supplementary material for: Tolfenamic Acid Derivatives: A New Class of Transcriptional Modulators with Potential Therapeutic Applications for Alzheimer’s Disease and Related Disorders
Source: Int J Mol Sci. 2023 Oct 16;24(20):15216. doi: 10.3390/ijms242015216 (PMC10607430; doi:10.3390/ijms242015216)
Supplement: Supplementary file 1 [file ijms-24-15216-s001.zip › ijms-2647164-supplementary.pdf]

Supplementary Materials

Tolfenamic Acid Derivatives: A new class of transcriptional modulators with potential therapeutic applications for Alzheimer's Disease and related disorders

Jaunetta Hill, Karim E. Shalaby, *et al.*
*Corresponding author. Email: nzawia@uri.edu

This PDF file includes:

Tables S1 to S3

Table S1. Enriched transcription factor (TF) binding motifs in promoter regions 300bp and 600bp upstream of genes with reduced (↓) or enhanced (↑) SP1-DNA binding following treatment with TA, TN3, or TN7 detected using ChIP-Seq

	↓			↑		
	**Motif**	**TF**	**TF family**	**Motif**	**TF**	**TF family**
**TA**	CG	KDM2B	CxxC	TTTTTAATTGGTT	NKX6-1	Homeodomain
	GCCTGAGG	TFAP2E	AP-2	TAATATT	ARID5A	ARID/BRIGHT
	GACACGTGC	HEYL	bHLH	TTACCAAA	CEBPA	bZIP
	CACGCG	HES4	bHLH	CATAATTACAT	POU2F1	Homeodomain, POU
	CACGTG	TCFL5	bHLH	ATCA	HDX	Homeodomain
	GGGG	ZNF202	C2H2 ZF	TAATT	SHOX	Homeodomain
	GGGCGTG	KLF7	C2H2 ZF	GTTAATTATTAACCAA	HNF1A	Homeodomain
	TGCGGG	ZBTB1	C2H2 ZF	TAATTACCATTATC	ALX1	Homeodomain
	GGGGGTGG	ZNF281	C2H2 ZF	ATTTGCATAT	POU2F2	Homeodomain, POU
	GGGGGGT	ZIC5	C2H2 ZF	GGTAATTATTAACC	HNF1A	Homeodomain
	CG	CGBP	CxxC	ATCAATCAA	PBX1	Homeodomain
	CG	MLL	CxxC	ATTGCAAAA	CEBPA	bZIP
	CCG	DNMT1	CxxC	AATTAGCATAGA	POU2F1	Homeodomain, POU
	CG	TET1	CxxC	GTTAATTATTAACC	HNF1A	Homeodomain
	GGCGCC	E2F2	E2F	TATA	TBPL2	TBP
	TGCGGG	GCM1	GCM	TTTAAT	ARID3C	ARID/BRIGHT
	TTTGCGCC	MYPOP	Myb/SANT	AT	HLX	Homeodomain
	ACGT	GMEB1	SAND	TGT	IRX4	Homeodomain
	GGGCAGCCAAGCGTGAC	PAX5	Paired box	TAAT	HOXC5	Homeodomain
	GGGCGGGAG	E2F1	E2F	TTATGTTAAATC	CDC5L	Myb/SANT
	CCGGAAG	ELK1	Ets	TATTAAT	ARID3B	ARID/BRIGHT
				ATT	SOX1	Sox
				TAAT	AC012531.1	Homeodomain
				TTTGAAT	LIN54	CxC
				TAAT	HOXD3	Homeodomain
				ATAAA	CDX4	Homeodomain
				TTTAT	HOXB9	Homeodomain
				CTTAATTG	NKX2-5	Homeodomain
				AT	DBX2	Homeodomain
				TAAT	HOXB4	Homeodomain
				ATCATAATATTT	FOXJ2	Forkhead
				GTAATTAA	HOXC9	Homeodomain
				ATATAAT	HMG20B	Sox
				TAAT	NKX6-3	Homeodomain
**TN3**	CG	KDM2B	CxxC	TAATATT	ARID5A	ARID/BRIGHT
	GCCTGAGG	TFAP2E	AP-2	TAATTACCATTATC	ALX1	Homeodomain
	GACACGTGC	HEYL	bHLH	ATTGT	SOX11	Sox
	CACGCG	HES4	bHLH	TTTTTAATTGGTT	NKX6-1	Homeodomain
	CACGTG	TCFL5	bHLH	ATTGT	SOX12	Sox
	GGGG	ZNF202	C2H2 ZF	TAAATAAATATTTC	FOXA2	Forkhead
	GGGCGTG	KLF7	C2H2 ZF	ACATA	FOXL1	Forkhead
	TGCGGG	ZBTB1	C2H2 ZF	AATTAGCATAGA	POU2F1	Homeodomain, POU
	GGGGGTGG	ZNF281	C2H2 ZF	GTTAATTATTAACC	HNF1A	Homeodomain
	GGGGGGT	ZIC5	C2H2 ZF	ATTAATTAAT	POU6F1	Homeodomain

	CG	CGBP	CxxC	TAATTA	LHX2	Homeodomain
	CG	MLL	CxxC	TTATGTTAAATC	CDC5L	Myb/SANT
	CCG	DNMT1	CxxC	TAA	TLX2	Homeodomain
	CG	TET1	CxxC	AT	HLX	Homeodomain
	GGCGCC	E2F2	E2F	TATTAAT	ARID3B	ARID/BRIGHT
	TGCGGG	GCM1	GCM	TTTAAT	ARID3C	ARID/BRIGHT
	TTTGCGCC	MYPOP	Myb/SANT	AT	DBX2	Homeodomain
	CCTGGCAAC	RFX6	RFX	TAAT	NKX6-3	Homeodomain
	ACGT	GMEB1	SAND	ATT	SOX1	Sox
	GCCTCAGGGCA	TFAP2A	AP-2	ATCATAATATTT	FOXJ2	Forkhead
	GGGCAGCCAAGCGTGAC	PAX5	Paired box	TCATTA	POU2F1	Homeodomain, POU
				TAAT	AC012531.1	Homeodomain
				TAATT	NOBOX	Homeodomain
				TAATT	LHX5	Homeodomain
				TAAT	HOXD3	Homeodomain
				TGT	IRX4	Homeodomain
				AA	HOXA2	Homeodomain
				TAAT	LHX4	Homeodomain
				AAACAAT	SRY	Sox
				TAAT	HOXC5	Homeodomain
				TAAT	HOXB4	Homeodomain
				ATATAAT	HMG20B	Sox
				ACGTAAACAAC	FOXO4	Forkhead
**TN7**	CG	KDM2B	CxxC	TAATATT	ARID5A	ARID/BRIGHT
	GCCTGAGG	TFAP2E	AP-2	ATATAAT	HMG20B	Sox
	GACACGTGC	HEYL	bHLH	TAAT	LHX4	Homeodomain
	CACGCG	HES4	bHLH	TTTTTAATTGGTT	NKX6-1	Homeodomain
	CACGTG	TCFL5	bHLH	TAATTACCATTATC	ALX1	Homeodomain
	GGGG	ZNF202	C2H2 ZF	AT	DBX2	Homeodomain
	GGGCGTG	KLF7	C2H2 ZF	ATTGCAAAA	CEBPA	bZIP
	TGCGGG	ZBTB1	C2H2 ZF	TAATT	NOBOX	Homeodomain
	GGGGGTGG	ZNF281	C2H2 ZF	ATTA	ZFHX3	Homeodomain
	GGGGGGT	ZIC5	C2H2 ZF	ATTGT	SOX12	Sox
	CG	CGBP	CxxC	CTAAAAATAG	MEF2A	MADS box
	CG	MLL	CxxC	ATTGT	SOX11	Sox
	CCG	DNMT1	CxxC	TGT	IRX4	Homeodomain
	CG	TET1	CxxC	AT	HLX	Homeodomain
	GGCGCC	E2F2	E2F	TATTAAT	ARID3B	ARID/BRIGHT
	TGCGGG	GCM1	GCM	TTTAAT	ARID3C	ARID/BRIGHT
	TTTGCGCC	MYPOP	Myb/SANT	ATT	SOX1	Sox
	CCTGGCAAC	RFX6	RFX	TAAATAAATATTTC	FOXA2	Forkhead
	ACGT	GMEB1	SAND	ATAAA	CDX4	Homeodomain
	GCCTCAGGGCA	TFAP2A	AP-2	TAAT	NKX6-3	Homeodomain
	GGGCAGCCAAGCGTGAC	PAX5	Paired box	ACGTAAACAAC	FOXO4	Forkhead
				ATGTAAACAACAA	FOXO3	Forkhead
				TATA	TBPL2	TBP
				TTTAT	HOXB9	Homeodomain
				AA	HOXA2	Homeodomain
				TAAT	AC012531.1	Homeodomain
				AAACAAT	SRY	Sox
				TTTGAAT	LIN54	CxC
				GTAATTAA	HOXC9	Homeodomain

ATCATAATATTT	FOXJ2	Forkhead
TTGTT	ZNF35	C2H2 ZF
TAAT	HOXC5	Homeodomain

Table S2. Enriched transcription factor (TF) binding motifs in promoter regions 300bp and 600bp upstream of differentially expressed genes following treatment with TA, TN3, or TN7 detected using mRNA-Seq or LC-MS-MS.

	**mRNAseq**			**LC-MS-MS**		
	**Motif**	**TF**	**TF family**	**Motif**	**TF**	**TF family**
**TA**	GGGGGCGGGG	SP1	C2H2 ZF	CG	TET1	CxxC
	GGGGGGGGGTGGTTTGGGG	RREB1	C2H2 ZF	CACGTG	TCFL5	bHLH
	GGGCAGCCAAGCGTGAC	PAX5	Paired box	ACGT	GMEB1	SAND
	CGAGGCGCAGTGATGCGTAGCGGC	PAX5	Paired box	GCGGGGGCGGGG	EGR1	C2H2 ZF
	CG	CGBP	CxxC	GGGGGCGGGGC	SP2	C2H2 ZF
	CCCCGGGC	TFAP2C	AP-2	CG	MLL	CxxC
	TGCGGG	ZBTB1	C2H2 ZF	CCG	DNMT1	CxxC
	TGCGGG	GCM1	GCM	CG	KDM2B	CxxC
	GGGGCCCAAGGGGG	PLAG1	C2H2 ZF	CG	CGBP	CxxC
	CCAATGGCGGTTGGGGG	MYB	Myb/SANT	GGGGGCGGGG	SP1	C2H2 ZF
	CACGTG	TCFL5	bHLH	ATGCGTGGGCGG	EGR4	C2H2 ZF
	GGCGGGAA	E2F4	E2F	TGCGTGGGCGT	EGR3	C2H2 ZF
	GGGCGGGAG	E2F1	E2F	GGCGGGAA	E2F4	E2F
	GGGG	ZNF202	C2H2 ZF	GGGCGGGAG	E2F1	E2F
	CCCCGGGC	TFAP2A	AP-2	GGCGCC	E2F2	E2F
	GGGGGGGGGCC	PATZ1	C2H2 ZF	GCGCAGGCGC	NRF1	Unknown
	TGTCAGGGGGC	INSM1	C2H2 ZF	TGCGTGGGCGT	EGR1	C2H2 ZF
	TGCGTGGGCGT	EGR3	C2H2 ZF	TGCGTGGGCG	EGR2	C2H2 ZF
	GGGGCGCAGCTGCGTCCC	NHLH1	bHLH	CACGCG	HES4	bHLH
	GCCCTGGGGC	TFAP2C	AP-2	GCGGACGTT	HINFP	C2H2 ZF
	CCG	DNMT1	CxxC	GGGCGGGAA	E2F6	E2F
	TGGCCACCAGGGGGCGC	CTCF	C2H2 ZF			
	GCCGAGGCCT	ZFY	C2H2 ZF			
	GGCGCC	E2F2	E2F			
	AGATCTCGCGAGA	ZBTB33	C2H2 ZF			
	GGGGGCGGGGC	SP2	C2H2 ZF			
	GGGCGGGGC	KLF5	C2H2 ZF			
	GGGCGTG	KLF7	C2H2 ZF			
	GCGGGGGCGGGG	EGR1	C2H2 ZF			
	CG	TET1	CxxC			

	TTGTT	ZNF35	C2H2 ZF			
	AAACAAACAAT	FOXD3	Forkhead			
	TAAACAAACA	FOXI1	Forkhead			
	GGGTGG	PAX4	Homeodomain, Paired box			
	AAACAAACATTC	FOXE1	Forkhead			
**TN3**	GGCGCC	E2F2	E2F	CACGTG	TCFL5	bHLH
	AGATCTCGCGAGA	ZBTB33	C2H2 ZF	CG	CGBP	CxxC
	GCGCAGGCGC	NRF1	Unknown	CG	KDM2B	CxxC
	CG	TET1	CxxC	CG	MLL	CxxC
	CACGTG	TCFL5	bHLH	CCG	DNMT1	CxxC
	CACGCG	HES4	bHLH	CG	TET1	CxxC
	ACGT	GMEB1	SAND	ACGT	GMEB1	SAND
	TTTGAAT	LIN54	CxC	GCGCAGGCGC	NRF1	Unknown
	CG	KDM2B	CxxC	GGGGGCGGGGC	SP2	C2H2 ZF
	TTTGCGCC	MYPOP	Myb/SANT	GGGCGGGAG	E2F1	E2F
	TTGGCGCGAAAATTG	E2F1	E2F	GGCGCC	E2F2	E2F
	TCGCGCCAAA	E2F1	E2F	GGCGGGAA	E2F4	E2F
	TGCGGG	ZBTB1	C2H2 ZF	GCGGACGTT	HINFP	C2H2 ZF
	GCCTCAGGGCA	TFAP2A	AP-2	GCGGGGGCGGGG	EGR1	C2H2 ZF
	GTGGGGA	MZF1	C2H2 ZF	CCGGAAG	ELK1	Ets
	GGGG	ZNF202	C2H2 ZF	GGGGGCGGGG	SP1	C2H2 ZF
	GGGGGGT	ZIC5	C2H2 ZF	CACGCG	HES4	bHLH
	CG	CGBP	CxxC	TGCGTGGGCGT	EGR3	C2H2 ZF
	CCG	DNMT1	CxxC	CCGGAAGTG	ELK4	Ets
	GGGGCCCAAGGGGG	PLAG1	C2H2 ZF	TGCGGG	ZBTB1	C2H2 ZF
	GGGCGGGGC	KLF5	C2H2 ZF	GGGCGTG	KLF7	C2H2 ZF
	CCCCGGGC	TFAP2A	AP-2	GGGCGGGAA	E2F6	E2F
	CCCCGGGC	TFAP2C	AP-2			
	GGGGGGGGGCC	PATZ1	C2H2 ZF			
	GCCTGAGG	TFAP2E	AP-2			
	GCGGGGGCGGGG	EGR1	C2H2 ZF			
	GGGGGTGG	ZNF281	C2H2 ZF			
	GGGGGCGGGGC	SP2	C2H2 ZF			
	CG	MLL	CxxC			
	GCCGAGGCCT	ZFY	C2H2 ZF			
	GGGGGCGGGG	SP1	C2H2 ZF			
	GCCCTGGGGC	TFAP2C	AP-2			
	GGGCGTG	KLF7	C2H2 ZF			
	GGGCGGGAG	E2F1	E2F			

	GGCGGGAA	E2F4	E2F			
**TN7**	GGGGGGGGGCC	PATZ1	C2H2 ZF	CG	TET1	CxxC
	GGGCGGGAA	E2F6	E2F	GCGCAGGCGC	NRF1	Unknown
	CACGTG	TCFL5	bHLH	ACGT	GMEB1	SAND
	CCG	DNMT1	CxxC	CG	MLL	CxxC
	GGCGGGAA	E2F4	E2F	CACGTG	TCFL5	bHLH
	GGGGCCCAAGGGGG	PLAG1	C2H2 ZF	GGCGCC	E2F2	E2F
	CG	CGBP	CxxC	TGCGTGGGCGT	EGR3	C2H2 ZF
	GGGGGCGGGGC	SP2	C2H2 ZF	CG	KDM2B	CxxC
	GGCGCC	E2F2	E2F	GGGGGCGGGGC	SP2	C2H2 ZF
	CCCCGGGC	TFAP2A	AP-2	TGCGGG	ZBTB1	C2H2 ZF
	CCCCGGGC	TFAP2C	AP-2	TGCGTGGGCG	EGR2	C2H2 ZF
	TGGCCACCAGGGGGCGC	CTCF	C2H2 ZF	TGCGTGGGCGT	EGR1	C2H2 ZF
	TGCGGG	ZBTB1	C2H2 ZF	CCG	DNMT1	CxxC
	GCGGGGGCGGGG	EGR1	C2H2 ZF	GGCGGGAA	E2F4	E2F
	GCGCAGGCGC	NRF1	Unknown	TACG	NAIF1	MADF
	GGGCGTG	KLF7	C2H2 ZF	CG	CGBP	CxxC
	CACGCG	HES4	bHLH	GCGGACGTT	HINFP	C2H2 ZF
	CG	TET1	CxxC	CACGCG	HES4	bHLH
	GGGCGGGAG	E2F1	E2F	AGATCTCGCGAGA	ZBTB33	C2H2 ZF
	GGGGGCGGGG	SP1	C2H2 ZF	ATGCGTGGGCGG	EGR4	C2H2 ZF
	TGTCAGGGGGC	INSM1	C2H2 ZF	GGGCGTG	KLF7	C2H2 ZF
	TGCGTGGGCGT	EGR3	C2H2 ZF	GCGGGGGCGGGG	EGR1	C2H2 ZF
	TAACGG	KLF9	C2H2 ZF	GGGCGGGAG	E2F1	E2F
	CGTA	HOXA3	Homeodomain	GGGGGCGGGG	SP1	C2H2 ZF
	TTATGTTAAATC	CDC5L	Myb/SANT			
	TTACATAA	NFIL3	bZIP			
	AAACAAACAAT	FOXD3	Forkhead			
	ACGT	ENSG00000250542	bZIP			
	TTACGTAA	ATF2	bZIP			
	CTTAATTG	NKX2-5	Homeodomain			
	AAACAAACATTC	FOXE1	Forkhead			
	GTAAT	EN1	Homeodomain			

TAGGAAATCGAAAGT	IRF7	IRF
CG	KDM2B	CxxC
TAATTG	BARHL1	Homeodomain
TAAATAAATATTTC	FOXA2	Forkhead
TTTGAAT	LIN54	CxC
TCGCGCCAAA	E2F1	E2F
TTGTT	ZNF35	C2H2 ZF
GATATGATAAGATA	MECOM	C2H2 ZF

Table S3. Neuronal pathways affected by treatment with TA, TN3 or TN7 and the affected genes from each treatment that belong to the pathway.

Pathway	TA	TN3	TN7
**Synaptogenesis Signaling Pathway**		ACTR2, AP2A2, AP2B1, AP2M1, ARPC2, ARPC3, CACNA2D1, CDC42, CFL1, CTNNB1, HSPA8, NAP1L1, NAP1L4, RAB5A, RAP1B, SNAP25	CDC42, CFL1, CTNNB1, HSPA8, MAPT
**Synaptic Long Term Potentiation**	GNA11, ITPR2, PDIA3, PPP3CA	GNA11, GNAQ, ITPR2, PPP1CA, PRKCA, RAP1B	
**Synaptic Long Term Depression**		CACNA2D1, GNA11, GNAI3, GNAQ, GNAS, ITPR2, PPP2CB, PPP2R1A, PRKCA, RAP1B, SMARCC2	GNAI3, GNAS, PPP2CB, PPP2R1A, PRDX6, PRKCA
**SNARE Signaling Pathway**		CACNA2D1, HSPA8, MYH14, RAB1A, RAB2A, SNAP25	
**Regulation of Actin-based Motility by Rho**	ACTA1, CDC42, CFL1, MYL12B, PFN1, RHOC	ACTA1, ACTR2, ARPC2, ARPC3, CDC42, CFL1, ITGB1, MYL12B, PFN1, RHOC	ACTA1, ARHGDIA, CDC42, CFL1, MYL12B, PFN1
**Reelin Signaling in Neurons**		ACTR2, ARHGEF40, ARPC2, ARPC3, CDC42, CFL1, ITGB1, RAP1B	
**Oxytocin In Brain Signaling Pathway**		CACNA2D1, GNA11, GNAI3, GNAQ, GNG2, ITPR2, PRKCA, RAP1B	GNAI3, GNB2, PRDX6, PRKCA
**Opioid Signaling Pathway**		AP2A2, AP2B1, AP2M1, CACNA2D1, CDC42, CTNNB1, GNA11, GNAI3, GNAQ, GNAS, GNG2, ITPR2, PRKCA, RAP1B	CDC42, CTNNB1, GNAI3, GNAS, GNB2, PRKCA
**Neurovascular Coupling Signaling Pathway**		CACNA2D1, GNAQ, ITPR2, SMARCC2	
**Myelination Signaling Pathway**	ARHGAP1, CDC42, FASN, HDAC2, LMNB1, PPP3CA	ARHGAP1, CDC42, CTNNB1, HDAC2, ITGB1, RAP1B	ARHGAP1, CDC42, CTNNB1, HDAC2

**GPCR-Mediated Nutrient Sensing in Enteroendocrine Cells**		CACNA2D1, GNA11, GNAI3, GNAQ, GNAS, GNG2, ITPR2, PRKCA	
**GPCR-Mediated Integration of Enteroendocrine Signaling Exemplified by an L Cell**		GNA11, GNAI3, GNAQ, GNAS, ITPR2	
**GNRH Signaling**		CACNA2D1, CDC42, GNA11, GNAI3, GNAQ, GNAS, GNG2, ITPR2, PRKCA, RAP1B	CDC42, GNAI3, GNAS, GNB2, PRKCA
**Ephrin Receptor Signaling**	ACP1, CDC42, CFL1, GNA11	ACP1, ACTR2, ARPC2, ARPC3, CDC42, CFL1, GNA11, GNAI3, GNAQ, GNAS, GNG2, ITGB1, RAP1B	CDC42, CFL1, GNAI3, GNAS, GNB2
**Ephrin B Signaling**	ACP1, CDC42, CFL1, GNA11, HNRNPK	ACP1, CAP1, CDC42, CFL1, CTNNB1, GNA11, GNAI3, GNAQ, GNAS, GNG2, HNRNPK	CAP1, CDC42, CFL1, CTNNB1, GNAI3, GNAS, GNB2, HNRNPK
**Endocannabinoid Neuronal Synapse Pathway**		CACNA2D1, GNA11, GNAI3, GNAQ, GNG2, ITPR2	
**Endocannabinoid Developing Neuron Pathway**		CTNNB1, GNAI3, GNG2, RAP1B	
**Dopamine-DARPP32 Feedback in cAMP Signaling**		CACNA2D1, GNAI3, GNAQ, GNAS, ITPR2, PPP1CA, PPP2CB, PPP2R1A, PRKCA	GNAI3, GNAS, PPP1CA, PPP2CB, PPP2R1A, PRKCA
**CREB Signaling in Neurons**		CACNA2D1, GNA11, GNAI3, GNAQ, GNAS, GNG2, ITPR2, PRKCA, RAP1B	
**Cholecystokinin/Gastrin-mediated Signaling**		CDC42, GNAQ, ITPR2, PRKCA, RAP1B, RHOC	
**CDK5 Signaling**		CAPN1, GNAS, ITGB1, PPP1CA, PPP2CB, PPP2R1A, RAP1B	GNAS, MAPT, PPP1CA, PPP2CB, PPP2R1A
**Agrin Interactions at Neuromuscular Junction**		ACTA1, CDC42, ITGB1, RAP1B	
